# Exploring SNPs in viral entry-related protein genes and their association with severe fever with thrombocytopenia syndrome virus infection risk

**DOI:** 10.1128/spectrum.03858-25

**Published:** 2026-07-15

**Authors:** Min Wang, Chengrui Ren, Guoqiang Deng, Yan Shen, Peng Cheng, Liqin Qian, Yong Qi, Weilong Tan, Yinchu Chai, Yuting Chen, Tian Zeng, Wen Yin, Chuanlong Zhu, Ming Yue

**Affiliations:** 1 Department of Infectious Diseases, The First Affiliated Hospital with Nanjing Medical University 74734, Nanjing, China; 2 General Office, Huangshan Center for Disease Control and Prevention, Huangshan, China; 3 Clinical Laboratory, Huangshan Center for Disease Control and Prevention, Huangshan, China; 4 Department of Infectious Disease Prevention and Control, Eastern Theater Command Centers for Disease Control and Prevention 725482, Nanjing, China; 5 Research and Production Center, Nanjing Shengxing Pest Control Technology Co., Ltd, Nanjing, China; 6 Key Laboratory of Infectious Diseases, Department of Epidemiology and Biostatistics, School of Public Health, Nanjing Medical University 12461, Nanjing, China; Shandong First Medical University, Jinan, Shandong, China

**Keywords:** severe fever with thrombocytopenia syndrome, severe fever with thrombocytopenia syndrome virus, cell receptors, DC-SIGN, DC-SIGNR, NMMHC-IIA, single nucleotide polymorphisms

## Abstract

Severe fever with thrombocytopenia syndrome virus (SFTSV), also known as Dabie bandavirus, is the pathogen causing severe fever with thrombocytopenia syndrome (SFTS). Human cell receptors are the first barriers to viral entry, proliferation, and host immune response. This study explored the association between single nucleotide polymorphisms (SNPs) in genes encoding receptors or related molecules that mediate SFTSV cell entry, such as dendritic cell-specific intercellular adhesion molecule-3-grabbing non-integrin (DC-SIGN), DC-SIGN-related protein (DC-SIGNR), and non-muscle myosin heavy chain IIA (NMMHC-IIA), and SFTSV infection susceptibility in the Chinese Han population, as well as the underlying potential biological mechanisms. From September 2020 to December 2022, 454 subjects from the First Affiliated Hospital with Nanjing Medical University were enrolled and divided into SFTSV-infected and uninfected groups based on SFTSV RNA and antibody test results. TaqMan SNP genotyping was performed on *DC-SIGN* (rs7248637, rs4804800, and rs11465421), *DC-SIGNR* (rs2277998), and *NMMHC-IIA* (rs2269529 and rs2269530). Logistic regression was used to assess the association between the candidate SNPs and SFTSV infection susceptibility, and the online bioinformatics tools were employed to explore potential biological functions of positive SNPs. Logistic regression analysis showed that individuals carrying the *DC-SIGNR* rs2277998 AA genotype were more susceptible to SFTSV infection, and *in silico* bioinformatics analysis generated the hypothesis that this locus might be involved in influencing gene transcription and expression, which warrants further functional validation. *DC-SIGNR* rs2277998 is significantly associated with SFTSV infection susceptibility in the Chinese Han population. The exact regulatory mechanisms require further experimental validation.

**IMPORTANCE:**

Severe fever with thrombocytopenia syndrome (SFTS) is an emerging infectious disease caused by severe fever with thrombocytopenia syndrome virus (SFTSV). Host genetic variation may modulate both disease susceptibility and severity by altering key functional proteins. Our study was the first to explore the association between single nucleotide polymorphisms (SNPs) in host genes encoding receptors or related molecules that mediate SFTSV cell entry, such as dendritic cell-specific intercellular adhesion molecule-3-grabbing non-integrin (DC-SIGN), DC-SIGN-related protein (DC-SIGNR), and non-muscle myosin heavy chain IIA (NMMHC-IIA), and SFTSV infection susceptibility in the Chinese Han population. The results showed that the homozygous mutation of *DC-SIGNR* rs2277998 AA genotype was associated with a significantly higher risk of SFTSV infection in the Chinese Han population, with *in silico* predictions pointing to a potential, yet unverified, role in regulating the *DC-SIGNR* gene transcription and expression, which may provide a new scientific basis for SFTSV prevention and treatment.

## INTRODUCTION

Severe fever with thrombocytopenia syndrome (SFTS), an acute infectious disease caused by severe fever with thrombocytopenia syndrome virus (SFTSV) and characterized by cytokine dysregulation and immune-mediated hyperinflammation, was first reported in the rural areas of Hubei and Henan provinces of China in 2009 (1, 2). Tick-to-human transmission is the primary route of human infection with SFTSV, and *Haemaphysalis longicornis* serve as the principal vector. In addition, animal-to-human and human-to-human transmissions have also been demonstrated by epidemiological studies (3). People are generally susceptible to SFTSV, and acute infection may result in long-term adverse health outcomes, with persistent abnormalities observed in a subset of convalescent individuals for platelet distribution width, mean platelet volume, mean corpuscular hemoglobin concentration, total protein, and albumin (4). A portion of the population has a high mortality rate. Currently, there are no approved vaccines or specific treatments available.

Viral entry into cells is essential for viral replication and proliferation in the human body. This process involves binding of the virus to specific receptors on target cells, followed by lipid membrane invagination and endocytosis. For SFTSV, its entry requires Gn/Gc binding to cell receptors and fusion with the cell membrane during endocytosis (5, 6). Meanwhile, conformational changes in viral proteins catalyze the fusion of the host endosome and virus membrane, promoting viral RNA into the cytoplasm (5, 7). However, receptors for SFTSV entry into cells remain unclear. To date, receptors or related molecules reported to mediate SFTSV entry include dendritic cell-specific intercellular adhesion molecule-3-grabbing non-integrin (DC-SIGN) (8), DC-SIGN-related protein (DC-SIGNR) (8), non-muscle myosin heavy chain IIA (NMMHC-IIA) (9), C-C motif chemokine receptor 2 (CCR2) (10), prolow-density lipoprotein receptor-related protein 1 (LRP1) (11), and receptor tyrosine kinase AXL (12). DC-SIGN, a calcium-dependent lectin expressed on dendritic cells and some tissue macrophages, interacts with mannose or oligosaccharide components on pathogen surfaces to promote pathogen cellular entry (13). DC-SIGNR, a type C lectin expressed on endothelial cells in the liver, lymph nodes, and placenta (14), has been reported to support SFTSV invasion into non-target cells (8). In addition, NMMHC-IIA, an actin-binding motor protein involved in cell migration, adhesion, polarization, and morphogenesis (15), also contributes to SFTSV entry by acting as an adhesion factor and promoting virus-cell membrane fusion (9). Moreover, SFTSV Gn can directly bind to CCR2 to enhance its cell entry, and CCR2 has been considered a host receptor for SFTSV (10). LRP1, a transmembrane receptor of the low-density lipoprotein receptor family, has been reported to mediate SFTSV infection via direct binding of its extracellular Cluster II and Cluster I domains to Gn (11). Recent genome-wide CRISPR activation screening identified a receptor tyrosine kinase AXL as a novel entry receptor for SFTSV (12). AXL, a member of the Tyro3, AXL, and MERTK family of receptor tyrosine kinases (16), is pivotal in the phagocytic clearance of apoptotic cells and the regulation of innate immunity. AXL-mediated SFTSV entry into cells utilizes the bridging action of growth arrest-specific protein 6 between AXL and phosphatidylserine on virus particles, triggering the activation of the PI3K-PLC signaling axis, driving macropinocytosis, ultimately facilitating SFTSV entry into cells (12).

It is widely acknowledged that the vast majority of human infectious diseases are the result of multifactorial effects (17). Host genetic variations can influence infectious disease progression and outcome; genetic variations of molecules related to SFTSV cell entry may affect individual susceptibility to SFTSV infection. Ding et al. (18) found that in Shandong Province, China, individuals carrying the A*02-B*15-DRB1*04 haplotype were linked to increased SFTSV infection risk, whereas those carrying the A*30-B*13-DRB1*07 haplotype had the opposite effect. Moreover, a Chinese case-control study reported that carriers of *platelet-derived growth factor-B* rs1800818 G allele were more susceptible to SFTSV infection (19). A cohort study revealed that the presence of *tumor necrosis factor-α* (*TNF-α*) rs1799964 C/rs361525 A alleles and multiple haplotypes were associated with decreased mortality risk in SFTS patients in the Chinese Han population, and the serum TNF-α levels were associated with the rs361525 A allele (20).

Given the above research background, our study aimed to explore the association between single nucleotide polymorphisms (SNPs) of host genes related to SFTSV cell entry and the susceptibility to SFTSV infection, as well as their potential biological mechanisms underlying this association, thereby providing new theoretical evidence for SFTSV prevention and treatment. Since the three aforementioned papers on cell receptors CCR2, LRP1, and AXL remained unpublished when this study was conducted, only *DC-SIGN*, *DC-SIGNR*, and *NMMHC-IIA* genes were included in this study, while *CCR2*, *LRP1*, and *AXL* genes were excluded. Further research plans to incorporate these three genes.

## RESULTS

### Demographic and clinical characteristics

A total of 454 participants were enrolled in this study. By examining medical data and investigating guardians, all subjects were confirmed to be genetically unrelated Chinese Han people, including 207 males and 247 females, with an average age of 58.1 ± 13.8 years. The SFTSV-infected group consisted of 244 cases, comprising 122 males and 122 females with an average age of 62.7 ± 11.9 years and 63.0 ± 10.6 years, respectively; the SFTSV-uninfected group included 210 cases, comprising 85 males and 125 females with an average age of 50.2 ± 14.6 years and 54.2 ± 14.2 years, respectively (Table S1). The two groups showed significant differences in age, gender, and the proportion of underlying diseases (*P* < 0.05) (Table S1). However, logistic regression was used later to adjust for the impact of these confounding variables. The minor allele frequency of all six SNPs was over 15%, and the genotype distributions in the SFTSV-uninfected group were in accordance with the Hardy-Weinberg equilibrium (HWE) (all *P* > 0.05) (Table 1).

**TABLE 1:** Sequences of primers and probes specific for the *DC-SIGN*, *DC-SIGNR,* and *NMMHC-IIA* SNPs^*a*^

Gene	SNPs (allele)	MAF^*b*^^,*c*^	*P^d^*	Primer and probe sequences (5′−3′)
*DC-SIGN*	rs7248637 (A > G)	0.324/0.273	0.748	Probe-G: FAM-AAATCCCAGTAAACC-MGB Probe-A: VIC-AAATCCCAATAAACCTG-MGB Forward primer: CAGGTAGTAGAGACAGAACTGTTCAGAGT Reverse primer: GCCTGGCCTATTATTTTTTGTAAGA
*DC-SIGN*	rs4804800 (G > A)	0.329/0.271	0.678	Probe-G: FAM-CTGTTCCTCGGTCTG-MGB Probe-A: VIC-AGTCCCTGTTCCTCAGT-MGB Forward primer: CCACGTGGTGTCCACTTCTTAA Reverse primer: AACGAAGCGTGCAAATCCA
*DC-SIGN*	rs11465421 (G > T)	0.238/0.288	0.971	Probe-A: FAM-CTTTATAATAAAATGGTAAACAT-MGB Probe-C: VIC-CTTTATAATAAACTGGTAAACAT-MGB Forward primer: ACAACTCGCCATTTTATTACACAAAG Reverse primer: CCCCGCCCCTCTAGTTGTAT
*DC-SIGNR*	rs2277998 (G > A)	0.176/0.162	0.094	Probe-G: FAM-CGGAACTGGCACGAC-MGB Probe-A: VIC-ACTGGCACAACTC-MGB Forward primer: CCAAGGACTGGACATTCTTCCA Reverse primer: CGAGCTGGGCCCTCACTT
*NMMHC-IIA*	rs2269529 (T > C)	0.417/0.375	0.326	Probe-T: FAM-CCGAGTCGATGTGC-MGB Probe-C: VIC-CCGAGTCGACGTGC-MGB Forward primer: AGCTTCCGCAGCTGTTTGAT Reverse primer: AGAGGAAGCAGCGCTCGAT
*NMMHC-IIA*	rs2269530 (C > T)	0.348/0.378	0.057	Probe-G: FAM-CCTGGAGGCGCAC-MGB Probe-T: VIC-CCTGGAGGCTCAC-MGB Forward primer: CCGCCCGGAAGAAGCT Reverse primer: TTCGTCCCGGTTCTTGTTG

^*a*^ SNPs, single nucleotide polymorphisms; DC-SIGN, dendritic cell-specific intercellular adhesion molecule-3-grabbing non-integrin; DC-SIGNR, DC-SIGN-related protein; NMMHC-IIA, non-muscle myosin heavy chain IIA; and MAF, minor allele frequency.

^*b*^ Minor allele frequencies in SFTSV-uninfected group.

^*c*^ Minor allele frequencies from HapMap of East Asia (EAS) (available at https://www.ncbi.nlm.nih.gov/snp).

^*d*^ *P* value of Hardy-Weinberg equilibrium for the SNPs among SFTSV-uninfected group.

### Association of the six SNPs with SFTSV infection susceptibility

The basic information of the six SNPs can be found in Table 2, and the genotype distribution of the six SNPs in the two groups is shown in Table 3. Four genetic models (co-dominant model, dominant model, additive model, and recessive model) were used for logistic regression analysis to assess the association between the six SNPs and SFTSV infection susceptibility. As shown in Table 3, after adjusting for gender, age, and underlying diseases, the result revealed that *DC-SIGNR* rs2277998 was associated with susceptibility to SFTSV infection. Specifically, compared to rs2277998 GG + GA genotypes, the AA genotype was associated with a significantly increased risk of SFTSV infection (recessive model: adjusted OR = 6.296, 95% CI = 1.693–23.411, *P* = 0.006), and the difference was still statistically significant after the multiple comparisons using Bonferroni adjustment (*P*_Bonferroni_ = 0.036). Furthermore, after adjusting for gender, age, and underlying diseases, the AA genotype remained significantly associated with increased risk of SFTSV infection in Firth’s penalized logistic regression analysis (recessive model: adjusted OR = 5.504, 95% CI = 1.596–18.977, *P* = 0.007). Additionally, no significant association was observed between the other five SNPs and SFTSV infection susceptibility (all *P* > 0.05).

**TABLE 2:** The basic information of the six SNPs^*a*^

Gene	SNPs (allele)	Region	Regulome DB scores	SNPinfo function		
				TFBS	Splicing	miRNA
*DC-SIGN*	rs7248637 (A > G)	3′-UTR	1f	/^*b*^	/	Y^*c*^
*DC-SIGN*	rs4804800 (G > A)	3′-UTR	1f	Y	/	Y
*DC-SIGN*	rs11465421 (G > T)	3′-UTR	1f	Y	/	/
*DC-SIGNR*	rs2277998 (G > A)	Exon	1f	/	Y	Y
*NMMHC-IIA*	rs2269529 (T > C)	Exon	4	/	Y	/
*NMMHC-IIA*	rs2269530 (C > T)	Exon	1f	/	Y	/

^*a*^ SNPs, single nucleotide polymorphisms; DC-SIGN, dendritic cell-specific intercellular adhesion molecule-3-grabbing non-integrin; DC-SIGNR, DC-SIGN-related protein; NMMHC-IIA, non-muscle myosin heavy chain IIA; and 3′-UTR, the three prime untranslated regions.

^*b*^ "/" indicates the absence of this function.

^*c*^ "Y" denotes the presence of this function.

**TABLE 3:** Association between six SNPs and SFTSV infection susceptibility^*a*^^,^^*b*^

SNPs (genotype)	SFTSV-infected group (*n* = 244) (%)	SFTSV-uninfected group (*n* = 210) (%)	OR^*c*^ (95% CI)	*P* value^*d*^	*P* value^*e*^	OR^*f*^ (95% CI)	*P* value^*g*^
*DC-SIGN* rs7248637							
GG	133 (54.51)	95 (45.24)	1.00	–^*h*^			
GA	95 (38.93)	94 (44.76)	0.759 (0.497–1.157)	0.200	1.00		
AA	16 (6.56)	21 (10.00)	0.573 (0.268–1.225)	0.151	0.906		
Dominant model			0.725 (0.484–1.085)	0.118	0.708		
Addition model			0.757 (0.552–1.040)	0.086	0.516		
Recessive model			0.651 (0.312–1.358)	0.253	1.00	0.658 (0.319–1.358)	0.257
*DC-SIGN* rs4804800							
AA	116 (47.54)	96 (45.71)	1.00	–			
AG	107 (43.85)	90 (42.86)	1.056 (0.691–1.614)	0.800	1.00		
GG	21 (8.61)	24 (11.43)	0.799 (0.394–1.620)	0.534	1.00		
Dominant model			1.004 (0.670–1.503)	0.985	1.00		
Addition model			0.951 (0.699–1.294)	0.750	1.00		
Recessive model			0.778 (0.396–1.527)	0.465	1.00	0.781 (0.401–1.523)	0.468
*DC-SIGN* rs11465421							
CC	139 (56.97)	122 (58.10)	1.00	–			
CA	85 (34.84)	76 (36.19)	0.993 (0.648–1.522)	0.974	1.00		
AA	20 (8.20)	12 (5.71)	1.935 (0.835–4.482)	0.123	0.738		
Dominant model			1.100 (0.733–1.652)	0.645	1.00		
Addition model			1.184 (0.856–1.638)	0.309	1.00		
Recessive model			1.941 (0.853–4.416)	0.114	0.684	1.895 (0.844–4.255)	0.121
*DC-SIGNR* rs2277998							
GG	167 (68.44)	139 (66.19)	1.00	–			
GA	60 (24.59)	68 (32.38)	0.688 (0.435–1.088)	0.110	0.66		
AA	17 (6.97)	3 (1.43)	5.605 (1.496–20.995)	0.011	0.066		
Dominant model			0.880 (0.571–1.357)	0.563	1.00		
Addition model			1.125 (0.787–1.607)	0.518	1.00		
Recessive model			6.296 (1.693–23.411)	**0.006**	**0.036**	**5.504 (1.596–18.977)**	**0.007**
*NMMHC-IIA* rs2269529							
CC	90 (36.89)	68 (32.38)	1.00	–			
CT	118 (48.36)	109 (51.90)	0.904 (0.580–1.410)	0.656	1.00		
TT	36 (14.75)	33 (15.71)	0.905 (0.492–1.667)	0.749	1.00		
Dominant model			0.904 (0.593–1.379)	0.640	1.00		
Addition model			0.942 (0.702–1.263)	0.688	1.00		
Recessive model			0.961 (0.554–1.667)	0.887	1.00	0.960 (0.556–1.657)	0.882
*NMMHC-IIA* rs2269530							
TT	127 (52.05)	93 (44.93)	1.00	–			
TG	82 (33.61)	82 (39.61)	0.734 (0.471–1.143)	0.171	1.00		
GG	35 (14.34)	32 (15.46)	0.748 (0.412–1.356)	0.338	1.00		
Dominant model			0.738 (0.492–1.107)	0.142	0.852		
Addition model			0.833 (0.630–1.102)	0.200	1.00		
Recessive model			0.857 (0.489–1.502)	0.590	1.00	0.857 (0.492–1.495)	0.587

^*a*^ SNPs, single nucleotide polymorphisms; SFTSV, severe fever with thrombocytopenia syndrome virus; DC-SIGN, dendritic cell-specific intercellular adhesion molecule-3-grabbing non-integrin; DC-SIGNR, DC-SIGN-related protein; NMMHC-IIA, non-muscle myosin heavy chain IIA; OR, odds ratio; and CI, confidence interval.

^*b*^ The logistic regression model adjusted for variables such as age, gender, and underlying diseases. Taking *DC-SIGN* rs7248637 as an example, A/G is a pair of alleles, and A is the less frequent gene, then co-dominant model: GA vs GG and AA vs GG; dominant model: GA + AA vs GG; additive model: AA vs GA vs GG; recessive model: AA vs GG + GA. Bold type indicates statistically significant results.

^*c*^ After adjusting for age, gender, and underlying diseases, the logistic regression model was used to calculate OR (95% CI) between the two groups.

^*d*^ After adjusting for age, gender, and underlying diseases, the logistic regression model was used to calculate the *P* value between the two groups*.*

^*e*^ The *P* value was corrected by Bonferroni*.*

^*f*^ After adjusting for age, gender, and underlying diseases, Firth's penalized logistic regression was used to calculate OR (95% CI) between the two groups (recessive genetic model).

^*g*^ After adjusting for age, gender, and underlying diseases, Firth's penalized logistic regression was used to calculate the *P* value between the two groups (recessive genetic model).

^*h*^ In the co-dominant model, when the wild-type homozygotes were set as the reference group, the OR was fixed at 1 by default; therefore, the *P* value is not applicable and is denoted by "−".

Based on the above-discovered association between *DC-SIGNR* rs2277998 and SFTSV susceptibility, we proceeded to explore the independent effect of rs2277998 by conducting a multiple-factor stepwise regression analysis. The baseline factors (age, gender, and underlying diseases), rs2277998 (recessive genetic model), and the other five SNPs were all included in this model, with the included variable having a *P* value of 0.050 and the excluded variable having a *P* value of 0.051. The result indicated that *DC-SIGNR* rs2277998 was an independent predictor of increased susceptibility to SFTSV infection (Table 4).

**TABLE 4:** Multivariate logistic regression analysis of factors influencing SFTSV infection susceptibility^*a*^

Variables	Regression coefficient	SE^*b*^	OR (95% CI)	*P* value
*DC-SIGNR* rs2277998 (AA vs GG + GA)	0.324	0.105	6.437 (1.731–23.933)	0.005
Age	0.014	0.002	1.065 (1.048–1.083)	<0.001
Gender	0.107	0.043	1.739 (1.152–2.625)	0.008

^*a*^ SFTSV, severe fever with thrombocytopenia syndrome virus; DC-SIGNR, DC-SIGN-related protein; OR, odds ratio; and CI, confidence interval.

^*b*^ The standard error of the stepwise regression model.

### Bioinformatics analysis of *DC-SIGNR* rs2277998

**Fig 1:**
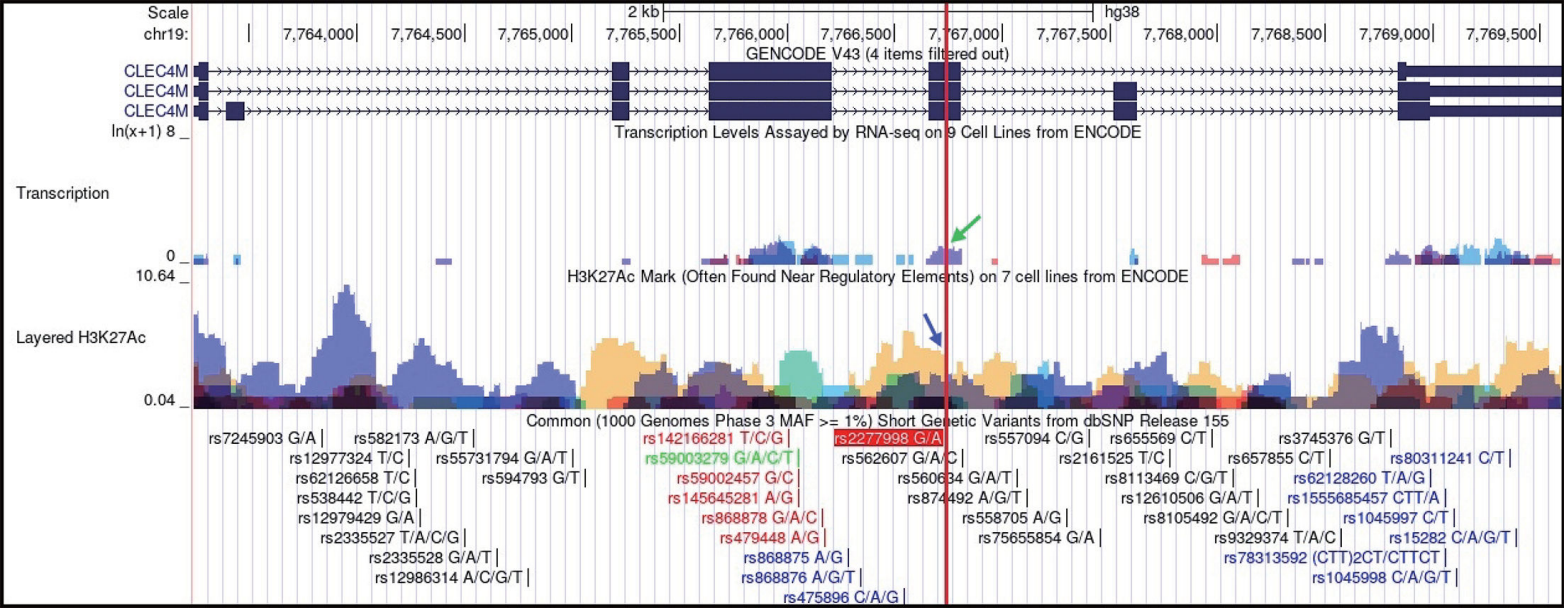
Functional annotation of *DC-SIGNR* rs2277998 based on UCSC gene browser. rs2277998 (marked by the red vertical line) is located within the transcription level regions detected by RNA-seq in nine cell lines (indicated by the green arrow) and within the active promoter element peaks (H3K27Ac histone mark) in seven cell lines (indicated by the blue arrow) (available at http://genome.ucsc.edu/).

The RegulomeDB, SNP Function Prediction, and UCSC Gene Browser websites were used to explore potential functional implications of *DC-SIGNR* rs2277998 that might underlie its association with SFTSV infection susceptibility. The RegulomeDB score of rs2277998 is 1f, indicating its potential functions like expression Quantitative Trait Loci (eQTL)/chromatin accessibility Quantitative Trait Loci (caQTL) and transcription factor (TF) binding/chromatin accessibility peak (Table 2; Table S2). Based on SNPinfo, rs2277998 is predicted to have splicing (exon splicing enhancer [ESE] or exon splicing silencer [ESS]) and miRNA-binding functions (Table 2). According to ENCODE and UCSC genome browser, as shown in Fig. 1, rs2277998 is located within regions exhibiting RNA-seq transcription levels in nine cell lines (GM12878, H1-hESC, HeLa-S3, HepG2, HSMM, HUVFC, K562, NHEK, and NHLF) and the peak of active promoter elements (H3K27Ac histone marker) in seven cell lines (GM12878, H1-hESC, HSMM, HUVFC, K562, NHEK, and NHLF). Thus, based strictly on these computational predictions, we hypothesize that the *DC-SIGNR* rs2277998 polymorphism may potentially mediate disease susceptibility by affecting gene transcription and expression processes, though direct experimental evidence is required to substantiate this claim.

## DISCUSSION

This study first explored the association between the SNPs of host genes related to SFTSV cell entry (*DC-SIGN*, *DC-SIGNR*, and *NMMHC-IIA*) and SFTSV infection susceptibility. The results showed that the homozygous mutation of *DC-SIGNR* rs2277998 AA genotype was significantly associated with an elevated risk of SFTSV infection in the Chinese Han population. Computational functional annotations suggest that this may be related to the mutation affecting the *DC-SIGNR* gene transcriptional expression, a hypothesis that remains to be experimentally verified.

*DC-SIGNR*, located on chromosome 19 and also known as *C-type lectin domain family 4 member M*, encodes DC-SIGNR, a lectin receptor expressed mainly on endothelial cells in lymph nodes and liver with a cytoplasmic domain, a transmembrane domain, a highly polymorphic neck region, and a carbohydrate recognition domain (21). The carbohydrate recognition domain is crucial for binding to glycans on the viral envelope. DC-SIGNR can recognize multiple pathogens, such as HIV, hepatitis C virus (HCV), severe acute respiratory syndrome coronavirus, *Mycobacterium tuberculosis*, and influenza A virus (14). During SFTSV infection, DC-SIGNR expression on non-target cells was reported to enhance SFTSV entry (8). The *DC-SIGNR* gene has seven exons spanning over 6.4 kbp (21). Exons 1–3 encode the cytoplasmic and transmembrane regions, exon 4 encodes the neck region, and exons 5–7 encode the ligand-binding domain. Among them, exons 4 and 5 exhibit polymorphisms, with exon 4 containing a variable number of tandem repeats and exon 5 containing a unique SNP (rs2277998), a G > A (Asp > Asn) missense mutation (21). Missense mutations, in which one amino acid is substituted by another, often lead to phenotypic consequences that are difficult to predict (22). A previous study on Thai HIV-seropositive individuals’ spouses found that females carrying *DC-SIGNR* heterozygous 7/5 and 9/5 repeat sequences and the rs2277998 A allele had a significantly reduced risk of HIV-1 infection (23). In our study, we found that *DC-SIGNR* rs2277998 AA was associated with an increased risk of SFTSV infection in the Chinese Han population. rs2277998 is located in exon 5 of *DC-SIGNR.* Based on SNPinfo, rs2277998 has splicing (ESE or ESS) and miRNA-binding functions, which may be related to alternative splicing of mRNA precursors of corresponding genes and may affect the transcription of mRNA by affecting miRNA binding, respectively. Additionally, according to the ENCODE database and UCSC Browser, rs2277998 lies on the peak of transcription level assayed by RNA-seq in nine cell lines and H3K27Ac histone marker in seven cell lines. Studies suggested potential pathogenic SNPs enriched in enhancer and regulatory element regions, particularly the H3K27Ac region, may impact gene transcription and expression, influencing disease susceptibility and prognosis (24, 25). Therefore, we hypothesize that *DC-SIGNR* rs2277998 may mediate host susceptibility to SFTSV infection by affecting gene transcription and expression. However, lacking genotype-stratified expression data in our cohort and given the current absence of DC-SIGNR eQTL data specific to the Chinese Han population in public databases, this putative regulatory mechanism remains predictive. Future studies will prioritize conducting genotype-expression association analyses within our study population, alongside rigorous *in vitro* and *in vivo* functional assays, to directly verify these regulatory effects.

*DC-SIGN* is also located on chromosome 19 and encodes DC-SIGN, a type II C-lectin receptor, which is mainly expressed on mature and immature dendritic cells of lymphoid tissues, such as lymph nodes, tonsils, and spleen, and can also be detected in gastrointestinal epithelial cells, macrophages in the placenta and lungs, and body fluids (26–28). DC-SIGN can recognize exogenous ligands with mannose and fucose residues, such as bacteria, fungi, and viruses, facilitating pathogen cell invasion (14, 29, 30). It also binds many endogenous ligands, promoting dendritic cell transendothelial migration and the formation of dendritic cell-T cell protrusions, thus boosting initial immune responses. This involvement affects the immune escape of various pathogenic microorganisms, such as HIV-1, HCV, *Mycobacterium tuberculosis*, and some parasites (31–34). Studies have linked *DC-SIGN* SNPs to susceptibility to certain pathogen infections. In the northern Chinese Han population, carriers of *DC-SIGN* rs735239 G and rs735240 A alleles and haplotype TC (*DC-SIGN* rs4804803 and rs2287886) showed higher fungal keratitis susceptibility (35). Sainz et al. (36) revealed that Caucasians carrying *DC-SIGN* rs4804800 G, rs11465384 T, rs7248637 A, and rs7252229 C alleles had significantly increased invasive pulmonary aspergillosis risk. Moreover, in African Colombians and Mestizos, those with *DC-SIGN* rs7248637 A allele were more prone to dengue fever (37). In addition, studies have also found a correlation between *DC-SIGN* SNPs and disease severity. A study in Irish women infected with HCV showed no association between rs4804803 in the *DC-SIGN* promoter region and the risk of HCV infection, while individuals carrying the rs4804803 G allele were more prone to severe liver disease outcomes (38). In another study on tuberculosis patients from four sub-Saharan African countries, carriers of the rs4804803 G allele had a significantly reduced risk of pulmonary cavity caused by *Mycobacterium tuberculosis* infection (39). In the Chinese eastern population, *DC-SIGN* rs735239 and rs4804803 were confirmed to have no association with pulmonary tuberculosis susceptibility, and carriers of the rs4804803 G allele had a lower proportion of fever but had no association with pulmonary cavities (40). Furthermore, *DC-SIGN* rs4804803 was associated with the occurrence of dengue fever and dengue hemorrhagic fever in Taiwan; further functional studies showed that the rs4804803 AG genotype increased cell surface DC-SIGN expression, leading to higher concentrations of TNF-α, IL-12p40, and IP-10, thereby enhancing immune response and reducing virus replication (41). Besides, in a study of patients with Russian forest encephalitis, when compared to the meningitis group, mild group, and control group, patients carrying *DC-SIGN* rs2287886 AA homozygotes and A allele had a significantly higher incidence of severe central nervous system disease (42). *DC-SIGN* rs7248637 and rs4804800 were also closely related to the severity of hand, foot, and mouth disease caused by EV71 in Chinese children (43). In our study, we investigated three *DC-SIGN* SNPs (rs7248637, rs4804800, and rs11465421), all located at 3′-UTR. However, no significant differences were found in these SNPs between SFTSV-infected and uninfected groups. This lack of significance might be due to the small sample size, which also prevented us from further analyzing the relationship between these SNPs and SFTSV infection severity and outcomes. Further research with a larger sample size is needed.

*NMMHC-IIA* is located on chromosome 22 and encodes NMMHC-IIA, which is involved in many cellular functional activities, including maintaining cell shape, cytoplasmic division, migration, and adhesion (44). According to previous reports, *NMMHC-IIA* gene polymorphisms were associated with the occurrence of various diseases, such as MYH9-related diseases, kidney diseases, hematological diseases, and cleft lip and palate (45–47). However, up to now, no studies have demonstrated the correlation between *NMMHC-IIA* gene polymorphisms and infectious diseases. In this study, two SNPs in the *NMMHC-IIA* gene (rs2269529 and rs2269530) were also confirmed to have no association with SFTSV infection susceptibility.

A unique strength of our study is that all uninfected controls had documented tick exposure or close contact with SFTS patients, rather than being selected only based on fever or thrombocytopenia, thereby markedly reducing selection bias. However, several research limitations should be mentioned. First, because the roles of CCR2, LRP1, and AXL as SFTSV receptors were identified after our study commenced, their SNPs were not included but warrant investigation in future combined analyses. Second, the inability to quantify viral exposure doses and the potential for asymptomatic infections could introduce misclassification bias in the control group. Third, the relatively small sample size—particularly the low frequency of the rs2277998 AA genotype—limited our ability to perform comprehensive stratified analyses and multicollinearity assessments. Consequently, the potential for residual confounding and unmeasured population stratification within the Chinese Han subgroups cannot be completely ruled out. Finally, as our findings currently lack functional validation and replication in an independent cohort, future large-scale, multicenter studies incorporating *in vitro* and *in vivo* mechanistic assays are essential to confirm the precise role of *DC-SIGNR* in SFTSV infection.

In summary, our study was the first to report the association between *DC-SIGNR* rs2277998 and SFTSV infection susceptibility among the Chinese Han population. This may provide a new scientific basis for SFTSV prevention and treatment.

## MATERIALS AND METHODS

### Study population

A total of 454 subjects from the First Affiliated Hospital with Nanjing Medical University between September 2020 and December 2022 were recruited in this study. All subjects were Chinese Han people from Jiangsu and Anhui provinces without blood relationship. The diagnosis criteria used followed “Consensus on the Diagnosis and Treatment of Severe Fever with Thrombocytopenia Syndrome (2022 edition)” (48). According to SFTSV RNA and IgM/IgG test results, subjects who signed the informed consent form were older than 18 years and were categorized into the following two groups: (i) SFTSV-infected group: subjects who had a history of working, living, or traveling in mountainous and hilly areas during the summer/autumn epidemic season, or were bitten by ticks, or had close contact with SFTSV-infected individuals within 2 weeks before onset; presented with an acute onset of illness, mainly characterized by fever (armpit temperature often above 38°C), accompanied by fatigue, poor appetite, nausea, vomiting, etc.; reduced white blood cell and/or platelet counts; and positive SFTSV RNA in peripheral blood; (ii) SFTSV-uninfected group: subjects who were bitten by ticks or exposed to SFTSV-infected individuals within the past month, with negative SFTSV RNA, IgM, and IgG in peripheral blood.

The exclusion criteria were as follows: (i) psychiatric patients; (ii) individuals with immune system diseases and/or long-term immunosuppressive therapy; (iii) HIV-infected individuals; and (iv) pregnant or lactating women.

### Clinical data collection

For the SFTSV-uninfected group, medical records from the 3 months before enrollment to 1 month after enrollment, including demographic data (gender, age, and occupation), underlying diseases, medication use, etc., were reviewed to exclude previous or asymptomatic SFTSV-infected individuals.

For the SFTSV-infected group, medical records such as demographics, underlying diseases, onset and consultation times, medical examination results, etc., were collected for SFTS diagnosis and subsequent inter-group comparison.

During the investigation process, a two-person dual verification system was used to input information, and patient privacy was protected.

### Research methods

A 5–10 mL fasting venous blood sample was collected from each subject using EDTA anticoagulant tubes and centrifuged at 1,761 × *g* for 5 min within 4 h. The obtained plasma and blood cells were stored in cryovials and frozen at −80°C for future use. The SFTSV RNA load was measured using SFTSV Nucleic acid Detection Kit (Sun Yat-sen University Da’an Gene Co., Ltd., China). SFTSV-specific IgM/IgG antibodies were tested by colloidal gold method using SFTSV Antibody Detection Kit (Nanjing Yuchen Biotechnology Co., Ltd., China). Human genomic DNA was extracted using the centrifugal column method (Blood Genomic DNA Extraction Kit; Beijing Tiangen Biochemical Technology Co., Ltd., China). Genotyping was performed by TaqMan probe real-time fluorescence quantitative PCR technology using the ABI Q3 real-time quantitative PCR instrument (Applied Biosystems, USA). The primer and probe sequences are shown in Table 1.

### *In silico* analysis

The NCBI dbSNP (https://www.ncbi.nlm.nih.gov/snp/) was utilized to view the specific location of the SNP locus on the chromosome and transcriptional regulation information. The RegulomeDB database (http://www.regulomedb.org/), the SNP Function Prediction (https://snpinfo.niehs.nih.gov/), and the UCSC Genome Browser (http://genome.ucsc.edu/) were used to predict the possible biological functions of positive SNPs.

### Quality control

Research quality was ensured through systematically trained operators, regular instrument calibration, random re-testing of 10% of samples, and a two-person dual verification system. The consistency rate of the repeated sample testing results was 100%, the success rate of genotyping was >94%, and all tests were performed in accordance with the manufacturer’s instructions.

### Statistical analysis

All statistical analyses were performed using SPSS (version 22.0), STATA (version 15.0), and RStudio (version 1.3.1093). The enumeration data and categorical variable data were compared using the chi-square (NPs and SFTSV infection susceptibilit²) test, while the normally distributed measurement data were compared using *t*-test. HWE was assessed in the SFTSV-uninfected group by the goodness-of-fit *χ*² test. Four genetic models (co-dominant model, dominant model, additive model, and recessive model) were used to explore the association of each SNP with SFTSV infection susceptibility. Taking *DC-SIGN* rs7248637 as an example, A/G is a pair of alleles, and A is the less frequent gene, then co-dominant model: GA vs GG and AA vs GG; dominant model: GA + AA vs GG; additive model: AA vs GA vs GG; and recessive model: AA vs GG + GA. Logistic regression adjusted for age, gender, and underlying diseases was performed to analyze the relationship between each SNP and SFTSV infection susceptibility by calculating the odds ratio and 95% confidence interval. Additionally, considering the low frequency of certain genotypes, Firth’s penalized logistic regression was employed. This method is particularly suitable for association analyses involving rare events or small sample sizes, as it effectively reduces the bias inherent in standard maximum likelihood estimation. The multiple-factor stepwise forward method was used to screen independent risk factors. All statistical analyses were two-sided, and *P* < 0.05 was considered statistically significant. Multiple comparisons were adjusted using Bonferroni correction, and the *P* value was adjusted to 0.008 (0.05/6).

## SUPPLEMENTAL MATERIAL

Tables S1 to S2 spectrum.03858-25-s0001.docx Table S1: Demographic and clinical characteristics of SFTSV-infected and uninfected groups. Table S2: RegulomeDB scores.

ASM does not own the copyrights to Supplemental Material that may be linked to, or accessed through, an article. The authors have granted ASM a non-exclusive, world-wide license to publish the Supplemental Material files. Please contact the corresponding author directly for reuse.
