# Supplementary material for: Exploring SNPs in viral entry-related protein genes and their association with severe fever with thrombocytopenia syndrome virus infection risk
Source: Microbiol Spectr. 2026 Jul 15;14(8):e03858-25. doi: 10.1128/spectrum.03858-25 (PMC13436344; doi:10.1128/spectrum.03858-25)
Supplement: Supplementary file 1 [file spectrum.03858-25-s0001.docx]

**TABLE S1.** Demographic and clinical characteristics of SFTSV infected and uninfected groups.

| Variables | SFTSV infected group  (n=244) | SFTSV uninfected group  (n=210) | *P-*value |
| --- | --- | --- | --- |
| Age (years) | 62.87±11.25 | 52.63±14.49 | < 0.001^a^ |
| ≤60, n (%) | 90 (36.9%) | 142 (67.6%) | < 0.001^b^ |
| Gender (male/female) | 122/122 | 85/125 | 0.042^b^ |
| underlying diseases ^c^, n (%) | 100 (41.0%) | 48 (22.9%) | < 0.001^b^ |

Abbreviations: SFTSV, Severe fever with thrombocytopenia syndrome virus.

^a^ Using *t*-test to compare normally distributed measurement data.

^b^ Using the χ^2^ test to compare enumeration data and categorical variable data.

^C^ Underlying diseases include hypertension, type 2 diabetes, coronary heart disease, old cerebral infarction, chronic obstructive pulmonary disease, chronic viral hepatitis, etc.

**TABLE S2.** RegulomeDB scores.

| Score | Supporting data |
| --- | --- |
| 1a | eQTL/caQTL + TF binding + matched TF motif + matched Footprint  + chromatin accessibility peak |
| 1b | eQTL/caQTL + TF binding + any motif + Footprint + chromatin accessibility peak |
| 1c | eQTL/caQTL + TF binding + matched TF motif + chromatin accessibility peak |
| 1d | eQTL/caQTL + TF binding + any motif + chromatin accessibility peak |
| 1e | eQTL/caQTL + TF binding + matched TF motif |
| 1f | eQTL/caQTL + TF binding/chromatin accessibility peak |
| 2a | TF binding + matched TF motif + matched Footprint + chromatin accessibility peak |
| 2b | TF binding + any motif + Footprint + chromatin accessibility peak |
| 2c | TF binding + matched TF motif + chromatin accessibility peak |
| 3a | TF binding + any motif + chromatin accessibility peak |
| 3b | TF binding + matched TF motif |
| 4 | TF binding + chromatin accessibility peak |
| 5 | TF binding or chromatin accessibility peak |
| 6 | Motif hit |
| 7 | Other |

Abbreviations: eQTL, expression Quantitative Trait Loci; caQTL, chromatin accessibility Quantitative Trait Loci; TF, transcription factor.

(available at https://www.regulomedb.org/regulome)
